# Physiological Action of the Hydrate of Chloral

**Published:** 1869-11

**Authors:** 


### ARTICLE VII.

### *Physiological Action of the Hydrate of Chloral.*

Dr. B. W. Richardson made an extremly interesting report on this subject to the Biological section of the British Association for the advancement of Science, at its recent meeting, from which we make the following extract : The hydrate of chloral, for the introduction of which into medical practice we are indebted to Liebreick (known for his researches on protagon), " is a white crystalline body, soluble in water, and yielding a solution not very disagreeable to the taste. It is made up by the addition of water to the substance chloral. Chloral, the composition of which is $C_2HCl_3O$, is the final product of the action of dry chlorine on ethylic alcohol. It is an oily fluid, thin, colorless, volatile, The specific gravity is 1.502 at 64° Fahr., and it boils at 202° Fahr. It has a vapor density of 73, taking hydrogen as unity. The odor is pungent. When chloral is treated with a little water, heat is evolved, and small stellate white crystals are formed as the fluid solidifies. The solid substance is the hydrate of chloral, $C_2HCl_3OH_2O$. The hydrate is slowly volatilized if it be exposed to the air, and the odor of it, were it not pungent, is so like melon as to be hardly distinguishable from melon. When heat is applied to the hydrate it distills over without undergoing decomposition.

"When to a watery solution of hydrate of chloral caustic soda or potassa is added, the hydrate is decomposed, chloroform ($CHCl_3$) is set free, and a formate of sodium or potassium, according to the alkali used, is formed. In was on a knowledge of this decomposition by an alkali that Liebreich was led to test the action of the substance physiologically. He conceived the idea that in the living blood the same change could be effected, and that the chloroform would be liberated so slowly that anæsthesia of a prolonged kind would result. To try this he subjected animals to the action of chloral, and even man, and proved that sleep could be rapidly induced without the second stage of excitement common to the action of chloroform when it is given by inhalation. Liebreich produced in a rabbit, by a dose of 0.5 gramme of the hydrate of chloral, a sleep which lasted nine hours. This dose was equivalent to 0.35 of chloral, and to 0.29 of chloroform. The symptoms, he found, were like those produced by chloroform. In some cases he gave the hydrate to the human subject. The first case was that of a lunatic, to whom he administered 1.35 gramme. No irritation was set up, and five hours of sleep was obtained. In a second case he gave internally a dose of 3.5 grammes to a man suffering from melancholia, by which he produced a sleep of sixteen hours.

"Such," said Dr. Richardson, "was an epitome of the facts placed before him at the time when he commenced to make his experiments. In setting out on his own account, he first prepared a standard solution of the hydrate. He found that 30 grains dissolved in 40 grains of water, and formed a satu[illegible]ted solution, the whole making up exactly the fluidrachm. The standard solution prepared in this way was very convenient.

"He next proceeded to inquire whether, by the addition of hydrate to fresh blood, chloroform was liberated. This was proved to be the fact ; the odor of chloroform was very distinct from the blood, and chloroform was itself distilled over from the blood, and condensed by cold into a receiver.

"The narcotic power of the hydrate was then tried on pigeons, rabbits, and frogs. The standard solution named above was employed, and [illegible] was administered either by the mouth or by hypodermic injection. The action was equally effective by both methods. The general results were confirmatory of Liebreich's own experience to a very considerable extent. They are as follows: In pigeons, weighing from 8½ to 11 ounces, narcotism was produced readily by the administration of from 1½ to 2½ grains of the hydrate. In these animals the dose of 2½ grains was the extreme that could be borne with safety, and a dose of 1½ grain was sufficient to produce sleep and insensibility. The full dose of 2½ grains produced drowsiness in a few minutes, and deep sleep with entire insensibility in twenty minutes. Before going to sleep there was in every case, whether the dose were large or small, vomiting. As the sleep and the insensibility came on, there was in every instance a fall of animal temperature, and even in cases where recovery followed, this decrease was often to the extent of five degrees. The respirations also fell in proportion, declining in one case from 34 to 19 in the minute during the stage of insensibility. From the full dose that could be borne by the pigeon the sleep which followed lasted from three and a half to four hours. Six hours at least was required for perfect recovery. During the first stages of narcotism in pigeons the evolution of chloroform by the breath was most distinctly marked.

"In rabbits weighing from 83 to 88 ounces, thirty grains of the hydrate were required in order to produce deep sleep and insensibility. A small dose caused drowsiness and want of power in the hinder extremities, but no distinct insensibility.

"When the full effect is produced in rabbits from the administration of the large dose, the drowsiness comes on in a few minutes: it is followed by want of power in the hinder limbs, and in fifteen minutes by deep sleep and complete insensibility. The pupil dilates and becomes irregular; the respiration falls (in one case from 60 to 36 in the minute) and the temperature declines 6° Fahr.; sensibility returns with the rise in number of respiratory movemonts, but in some cases falls again during the process of recovery. The drowsiness, or, if the animal is left alone, what may be called sleep, lasts from five and a half to six hours. But it was observed that the period of actual anæsthesia was very short, lasting not longer than half an hour, after which the skin seemed rather more than naturally sesitive to touch. During recovery there are tremors of muscles almost like the rigors from cold; they are due probably to great failure of animal temperature.

"In frogs a grain of the hydrate causes almost instant insensibility, coma, and death.

"In further prosecution of his research, the author tested on similar subjects, the effect of chloroform, bichloride of methylene, tetrachloride of carbon, and chloride of amyl. In all the observations with these substances, the narcotizing agent was used by hypodermic injection. It was found, as a result of these inquiries, that seven grains of chloroform, five of tetrachloride of carbon, and seven of chloride of amyl, produced the same physiological effect as two grains of the hydrate. Seven grains of bichloride of methylene induced a shorter insensibility. A rabbit subjected to thirty grains of chloroform slept four hours and twenty-five minutes; and a pigeon subjected to seven grains slept three hours and twenty-five minutes. All these agents caused vomiting in birds, before the insensibility was pronounced, the same as did the hydrate; but in no animal was there any sign of the stage of excitement which is seen when the same agents are administered by inhalation. This fact is most important as indicating the difference of action of the same remedy by difference in the mode of administration.

The temperature of the body was reduced by the agents named above, but not so determinately as by the hydrate.

"Two animals, pigeons, made to go into profound sleep, the one by the hydrate, the other by chloroform (each substance administered subcutaneously), were placed together, and the symptoms were compared. The sleep from the chloroform was calmer; there was freedom from convulsive tremors, which were present in the animal under the hydrate, and recovery was, it was thought, steadier. It was observed, and the fact is well worthy of note, that no irritation was caused in the skin or subjacent parts by the injection of the chloroform and other chlorides.

"The neutralizing action of the hydrate on strychnia was tried, and it was determined that the substance arrests the development of the tetanic action of the poison for a short period, and maintains life a little longer afterwards, but does not avert death. This subject deserves further elucidation.

"When the hydrate of chloral is given in an excessive dose it kills; there are continuance of sleep, convulsion, and a fall of temperature of full eight degrees before death.

"The post mortem appearances were noticed after a poisonous dose. The vessels of the brain are found turgid with blood. The blood is fluid, and coagulation is delayed (in a bird to a period of three minutes), but afterwards a loose coagulum is formed. The color of the brain substance is darkish pink. The muscles generally contain a large quantity of blood, which exudes from them, on incision, freely. This blood coagulates with moderate firmness. Immediately after death all motion of the heart is found to be arrested. The organ is left with blood on both sides, but with more in the right than in the left side. The color of the blood on the two sides is natural, and the coagulation of this blood is moderately firm. The other organs of the body are natural.

"Other observations were made on the changes which the blood undergoes when the hydrate of chloral is added to it. The corpuscles undergo shrinking, and are crenate; and when excess of hydrate is added the blood is decomposed in the same way as when treated with formic acid. The summary of the author's work may be put as follows;—

"Hydrate of chloral, administered by the mouth or by hypodermic injection, produces, as Liebreich states, prolonged sleep.

"The sleep it induces, as Liebreich also shows, is not preceded by the stage of excitement so well known when chloroform is administered by inhalation.

"The narcotic condition is due to the chloroform liberated from the hydrate in the organism, and all the narcotic effects are identical with those caused by chloroform.

"In birds the hydrate produces vomiting in the same manner, and to as full a degree, as does chloroform itself.

"The sleep produced by hydrate of chloral is prolonged and during the sleep there is a period of perfect anæsthesia; but this stage is comparatively of short duration.

"The action of the hydrate is (as Liebreich assumes) first on the volitional centres of the cerebrum; next on the cord; and, lastly, on the heart.

"*Practical applications.*—Whether hydrate of chloral will replace opium and the other narcotics is a point on which the author was not prepared to speak. It is not probable that it will supersede the volatile anæsthetics for the purpose of removing pain during the performance of surgical operations, but it might be employed to obtain and keep up the sleep in cases of painful disease. This research had, however, led to the fact that chloroform, when injected subcutaneously in efficient doses, leads to as perfect and as prolonged a narcotism as the hydrate, with an absence of other symptoms caused by the hydrate, and which are unfavorable to its action. This was a new truth in regard to chloroform, and might place it favorably by the side of the hydrate for hypodermic use. Lastly, as the hydrate acts by causing a decomposition of the blood—*i. e.*, by undergoing decomposition itself and seizing the natural alkali of the blood, it adds to the blood the formate of sodium. How far this is useful or injurious remains to be discovered. But while putting these views as to practical application at once and fairly forward, Dr. Richardson said it was due to Liebreich to add that his (Liebreich's) theory and his experiments have done fine service in a physiological point of view. They have shown in one decisive instance that a given chemical substance is decomposed in the living body by virtue of pure chemical change, and that the symptoms produced are caused by one of the products of that decomposition. The knowledge thus definitely obtained admits of being applied over and over again in the course of therapeutical inquiry."—*Med. Times and Gazette.*

